# Numerical analysis of the precursory information of slope instability process with constant resistance bolt

**DOI:** 10.1038/s41598-021-01387-z

**Published:** 2021-11-08

**Authors:** Feng Chen, Xue-bin Wang, Yan-hong Du, Chun-an Tang

**Affiliations:** 1 School of Mechanics and Engineering, Liaoning Technical University, Fuxin, 123000 Liaoning China; 2 Institute of Computational Mechanics, Liaoning Technical University, Fuxin, 123000 Liaoning China; 3 State Key Laboratory for Geomechanics and Deep Underground Engineering, China University of Mining and Technology, Beijing, 100083 China; 4 School of Civil Engineering, Dalian University of Technology, Dalian, 116024 China

**Keywords:** Natural hazards, Engineering

## Abstract

The instability of slope has already threatened life and property safety of the people, and improving the monitoring method of slope stability has important theoretical and practical significance for disaster prevention and reduction. According to the idea of “Newton force sudden drop and catastrophic occurrence” proposed by M.C. He in the landslide monitoring, a numerical model with constant resistance bolt has been established. Through numerical simulation research, it is found that the maximum principal stress, minimum principal stress and shear stress of the intersection point P of landslide surface and constant resistance bolt are sudden growth and sudden decrease, the vertical displacement and lateral displacement of this point P appear rise and fall before three kinds of stress. When loading to the next step of the step where three stress have reduced to a minimum value the slope is unstable and destroyed. At this time, the constant resistance bolt has undergone larger plastic deformation and damaged. Finally, comparing the stress curves and the acoustic emission (AE) curves, it can be seen that stress decreases from the maximum value and the AE curves begin to show a significant rise, the two curves display opposite law. It can be seen from the AE diagram that the failure mode of the slope is a combined tension and shear failure. The numerical test results provide a new idea for real-time monitoring and forecasting of slope.

## Introduction

**Figure 1:**
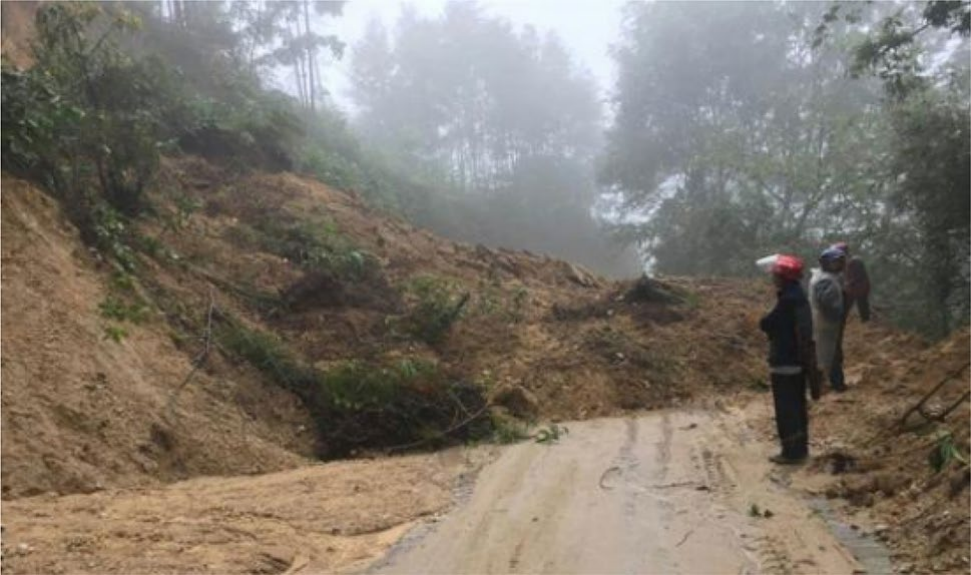
Mountain landslide blocking road.

Landslide is a phenomenon that extensive soils, plenty of rock masses depositing on the slope slide down along a certain sliding surface^1,2^. It often blocks traffic, destroys factories and mines, stops up rivers, destroys villages and farmland, causing huge economic losses and casualties^3,4^. If the stress is too large and the strength is too low inside the slope, part of the rock mass will slide downward, which is the root cause of the slope slippage. Its damage characteristics are shown in Fig. 1. Therefore, real-time effective monitoring and forecasting of landslide disasters is very important for disaster prevention and mitigation.

With the continuous development of science and technology, the monitoring and forecasting methods of landslide disasters have been improved by a large margin than before. At present, slope monitoring methods can be roughly divided into multiple types of monitoring technologies that use surface displacement, groundwater pressure, blasting vibration, anchoring stress, and deep displacement as the monitoring quantities^5–7^. With the rapid development of electronic information technology and the cross integration of various disciplines, different monitoring technologies such as photogrammetry, three-dimensional laser scanning, interferometric synthetic aperture radar (InSAR), measurement robots, automated monitoring network, optical fiber sensing, and microseismic monitoring continue to emerge^8–12^. Scholars all over the world have made full use of various available means to monitor landslides and have achieved abundant results^13,14^. Hu et al. studied the fractal characteristics of the track curve of landslide surface monitoring points, and analyzed the movement direction change process of each point from the stage of landslide evolution^15^. Wang et al. had applied GPS precise point positioning technology to real-time dynamic monitoring and early warning of geological disasters such as landslides^16^. Wang et al. pointed out that differential synthetic aperture radar interferometry technology can be used to monitor large-scale landslides^17^. Keefer et al. developed a real-time monitoring system for the San Francisco Bay Area in California based on empirical and theoretical relationship between rainfall and landslide to issue landslide warnings during major storms^18^. Uchimura et al. had used tilt sensors to predict and warn of surface damage on slopes^19^. Xu et al. introduced 3D laser scanning technology into the monitoring and analysis of landslide deformation, making full use of a large number of natural features on the landslide body as monitoring points to completely monitor and analyze slope deformation^20^. Xue et al. established a landslide deformation analysis model based on DEM grid to study the deformation between Heifangtai landslide in Yongjing County, Gansu Province from 1977 to 2010 and forecast the future trend of landslide^21^. The above-mentioned various monitoring methods make the landslide warning technology more intelligent, visualized and simplified. However, the research on the application of bolt in slope monitoring is not in-depth.

In recent years, with the rapid development of computer science and computational mechanics, numerical simulation technology has increasingly become a reliable and effective research method. Common numerical simulation methods can be divided into two main types: continuous medium method and discontinuous medium method. Continuum methods include finite element method (FEM) and extended finite element method/generalized finite element method (XFEM/GFEM), finite difference/finite volume method (FDM/FVM), boundary element method (BEM), real failure process analysis method (RFPA) and so on^22–24^. In order to better analyze the mechanical behavior of the rock block system cut by natural joints and fractures, scholars have proposed a series of discontinuous media methods, such as discrete element method (DEM)/particle discrete element method (PFC), discrete fracture network grid method (DFN)^25–27^. Among the above numerical simulation methods, only the RFPA software considers the heterogeneity of the rock.

In order to solve above problems, M.C. He had proposed a two-body catastrophic mechanics theory of geological hazards based on the detection method of Newton force variation^28^. By using a self-developed remote monitoring and early warning system of Newton force change, the scientific phenomenon of "Newton force sudden drop and landslide occurrence" was confirmed through on-site monitoring of landslide, and the leap from "phenomenal monitoring" to "essential monitoring" had been realized^28^. Based on above idea of M.C. He, this paper uses RFPA-Centrifuge software to establish a slope model with constant resistance bolt, and the conclusion that “the sudden drop in stress and displacement means the occurrence of landslide” is drawn.

## RFPA-centrifuge basic principles

Centrifugal loading test is to increase volume force of geotechnical model and form artificial gravity by high-speed rotation of centrifuge, which reflects the mechanical characteristics of engineering prototype, observes the failure mode and understands the safety reserve coefficient. In this RFPA-Centrifuge software, the self-weight of model element is gradually increased, and the slope is considered to be unstable when the fracture surface runs through.

Assuming that the geometric size of the model is $1/n$ times that of the realistic prototype, The bulk density of realistic prototype is $\gamma_{r} = \rho g$, *ρ* is soil mass quality, *g* is gravity acceleration, The bulk density of model is $\gamma_{m} = \rho a$, *a* is gravity acceleration of model. According to the condition that the stress of the model is consistent with that of realistic prototype, namely $\sigma_{r} = \sigma_{m}$, we can obtain $\rho gh_{r} = \rho ah_{m}$, *h*_*r*_ is realistic prototype geometric dimension, *h*_*m*_ is model geometric dimension. Therefore:

1 $$a = (h_{r} /h_{m} )g = ng$$

The maximum number of failure elements in RFPA-Centrifuge is used as a criterion for slope instability. In the calculation and analysis of RFPA, the RFPA centrifugal loading method automatically records the number of destroy elements in each loading step, and uses this simple and effective method to judge slope instability.

Safety factor, also known as stability factor, is an important concept in the study of slope stability. The safety factor of traditional slope stability analysis is defined as the ratio of sliding resistance to sliding force of sliding surface. The safety reserve coefficient that is characterized by *K* has been defined as the ratio of the element own weight to the initial element own weight at the time of instability. The calculation formula is as follows:

2 $$K = (\gamma + \gamma (S_{step} - 1)\Delta g)/\gamma = 1 + (S_{step} - 1)\Delta g$$

where *K* is safety coefficient, *S*_*step*_ is number of loading steps when the slope is destroyed, Δ*g* is centrifugal loading coefficient, namely the percentage of self-weight increase in each step of calculation. *γ* is element bulk density.

**Figure 2:**
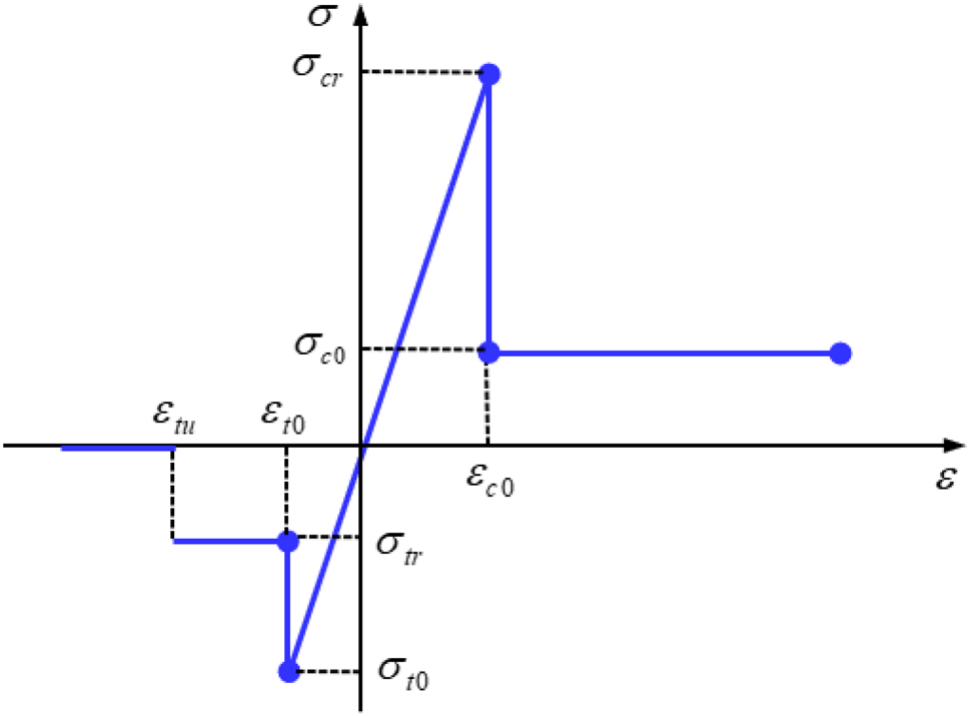
The elastic-brittle constitutive laws of element.

RFPA-Centrifuge adopts a constitutive relationship with residual strength. The elastic damage constitutive relation of element is shown in Fig. 2. When the stress or strain states in the element meet a given threshold, the element begins to accumulate damage. The cumulative damage process of element elastic modulus can be given by

3 $$E = (1 - D)E_{0}$$

where D is the damage variable; *E* and *E*_*0*_ are elastic modulus before and after the element damage, respectively.

The equation of the damage variable under uniaxial tension can be expressed as:

4 $$D = \left\{ {\begin{array}{ll} 0 & {\varepsilon < \varepsilon_{t0} } \\ {1 - \lambda \varepsilon_{t0} /\varepsilon } & {\varepsilon_{t0} < \varepsilon < \varepsilon_{tu} } \\ 1 & {\varepsilon > \varepsilon_{tu} } \\ \end{array} } \right.$$

where $\lambda$ is element residual tensile strength coefficient, i.e. $\sigma_{tr}$ = *λ* $\sigma_{t0}$; $\varepsilon_{t0}$ is the tensile strain corresponding to the elastic limit state of element, and it is also the initial damage threshold of element; $\varepsilon_{tu}$ is the ultimate tensile strain of element.

## Constant resistance bolt introduction

**Figure 3:**
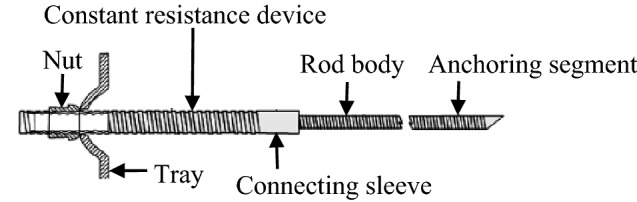
Structure of constant resistance bolt.

When large deformation and instability destruction occurs in slopes, tunnel surrounding rocks, etc., traditional rigid bolt is frequently broken due to its small deformation and cannot adapt to large deformation failure^29^. In order to solve such problems, M.C. He team had independently developed a constant resistance bolt with constant working resistance and stable large deformation characteristics (as seen in Fig. 3). This bolt is mainly composed of a nut, a tray, a constant resistance device and a rod body^30,31^. Among them, the constant resistance device comprises a constant resistance sleeve and a constant resistance body, and the inner surface of the constant resistance sleeve and the outer surface of the constant resistance body are both threaded structures, which reduces the weight of a bolt to a certain extent. The constant resistance bolt has been successfully applied to the surrounding rock support of soft rock roadway and deep roadway, which can effectively control geological disasters such as rockburst^32^.

## Numerical model

**Figure 4:**
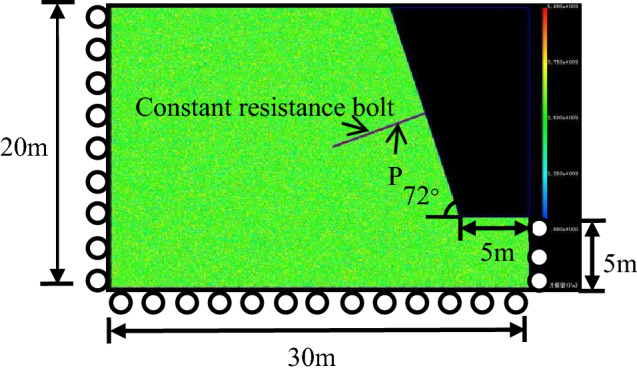
Numerical model of slope with constant resistance bolt.

In this paper, RFPA-Centrifuge software is used to analyze stress state of constant resistance bolt in the process of slope instability. The acceleration coefficient of gravity is 0.01. The lower and bilateral boundaries of this model are set as normal constraint and the upper boundary is set as free boundary. The slope angle is 72° and the slope height is 15 m. Other various dimensions of this numerical model are shown in Fig. 4. The failure of the rock follows the Mohr–Coulomb failure criterion, the mechanical parameters of the units are assumed to be assigned according to a Weibull distribution, the constitutive relationship of the rock adopts the elastic-brittle constitutive relationship with residual strength.

**Figure 5:**
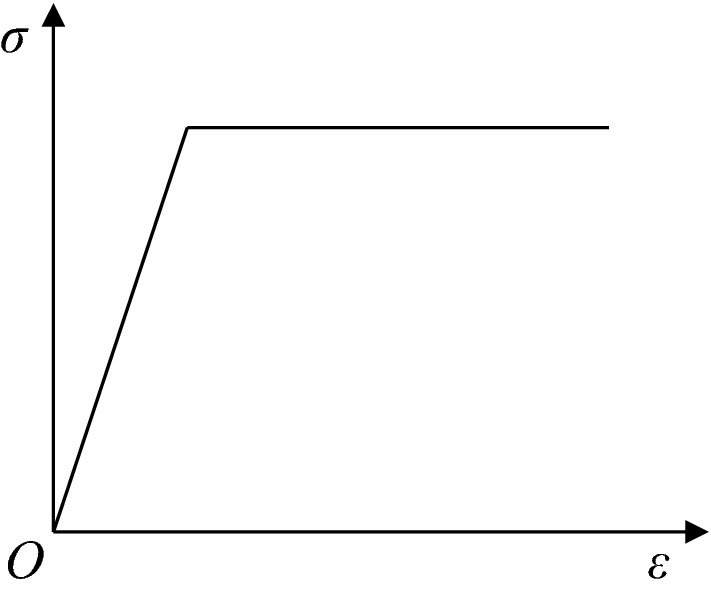
Elastic–plastic constitutive relation of constant resistance bolt.

Constant resistance bolt is inserted into slope deep part, and its length is 7.5 m to ensure the bolt passes through potential sliding surface in this paper. Point P in Fig. 3 is the intersection of slope sliding surface and constant resistance bolt, and it is located on the constant resistance bolt. Because the constant resistance bolt deforms considerably to maintain a certain working stress and has a time-dependent mechanical behaviour, the constitutive relation is simplified as an ideal elastic–plastic model, as shown in Fig. 5. Numerical calculation is processed according to the plane strain problem. The calculation parameters of the numerical model are shown in Table 1.

**Table 1:** The calculation parameters of numerical mode.

Parameter	Rock	Constant resistance bolt
Young’s modulus (GPa)	21.3	210
Friction angle (°)	32.7	30
Uniaxial compressive strength (MPa)	165	350
Density (kg/m^3^)	2500	7800
Homogeneity index	3	100
Poisson’s ratio	0.24	0.27

## Numerical calculation results and analysis

### Point P stress analysis

**Figure 6:**
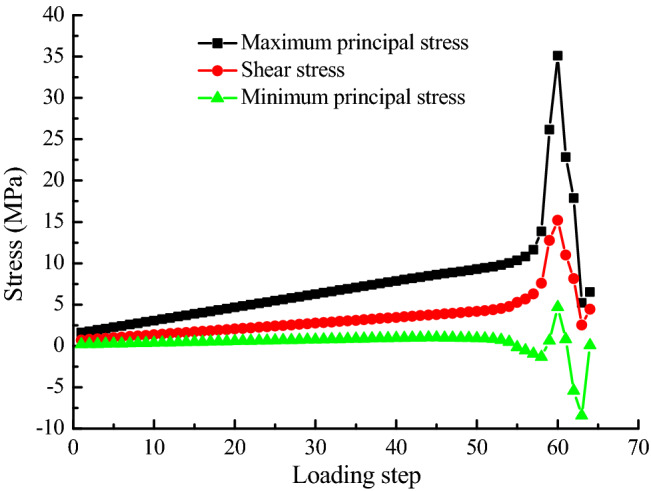
Various stress curve of point P in landslide process.

**Figure 7:**
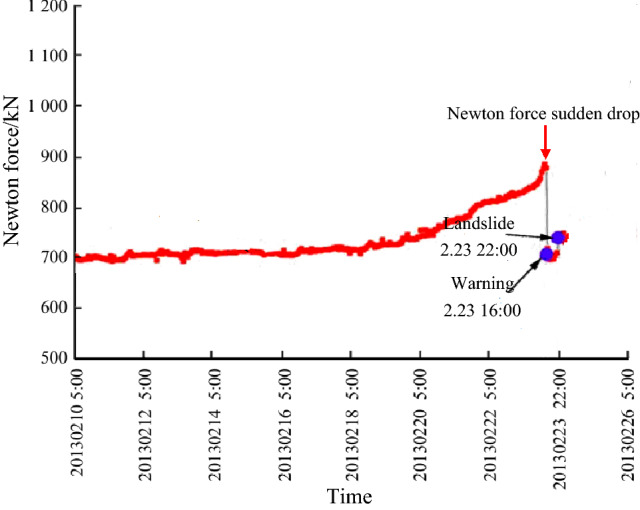
Monitoring warning curves of Newton force in Nanfen open pit mine.

Figure 6 shows the variation of maximum principal stress, shear stress and minimum principal stress at point P on constant resistance bolt along with loading step in the process of slope instability. That is to say, the stress and displacement at this point can reflect the dynamic change process of landslide. As can be seen from the figure that above three stress curves all have the characteristics of sudden rise and sudden drop with the increase of loading step, but the increase rate of stress value is relatively slow in the early stage of loading. The change trend of stress curve obtained by numerical simulation is very similar to the field monitoring results (as shown in Fig. 7), which also indicates that the numerical test method is accuracy and feasibility. The specific evolution process is described below.

**Figure 8:**
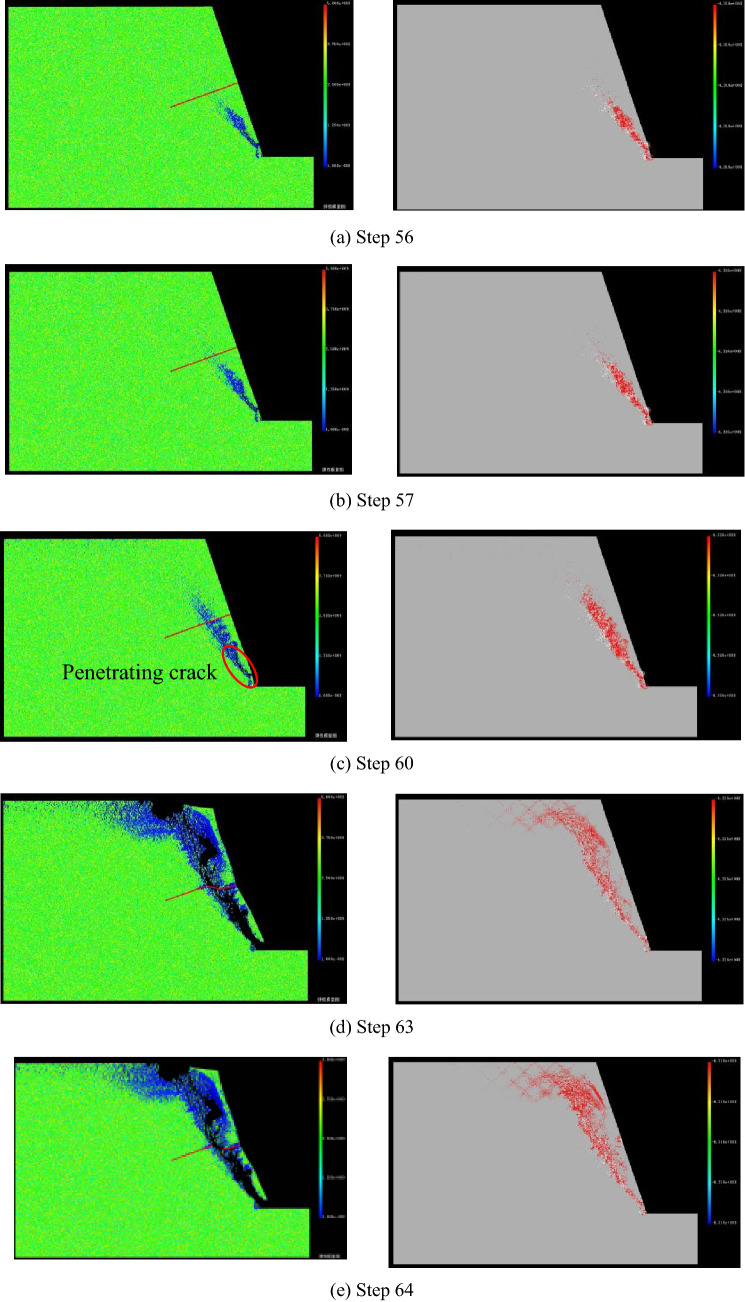
Landslide process (The left column is the elastic modulus diagram, the right column is the acoustic emission diagram).

1. Before loading to step 57, the maximum principal stress and shear stress increase linearly, the slope of straight line is low, and the growth rate of two stress values is slow. Before loading to step 53, the minimum principal stress curve is roughly a horizontal straight line. Subsequently, there is a small amplitude fall, and a minimum value occurs at step 56. At the time, the cracks on slope gradually begin to emerge upward from slope foot (see Fig. 8a).
2. From the 57th step, the maximum principal stress, shear stress and minimum principal stress appear to increase significantly, and all reach the maximum value of 35.08 MPa, 15.2 MPa and 4.68 MPa at step 60 with increments of 23.43 MPa, 8.91 MPa, 5.62 MPa, respectively. There is a obvious crack coalescence phenomenon in the slope (see Fig. 8c), and there is no obvious bending change in the shape of constant resistance bolt during this stage.
3. With the continuous loading, the maximum principal stress, shear stress and minimum principal stress all show a significant downward trend. When loading to the 63rd step, above three stress minimum values that are 5.19 MPa, 2.54 MPa, − 8.42 MPa appear in turn, and the decreasing amplitude is 29.89 MPa, 12.66 MPa, and 13.1 MPa in proper sequence. It can be seen from Fig. 8d that the deformation of constant resistance bolt is obvious along with the increase of slope crack length, and obvious plastic deformation occurs, but there is no breakage failure.
4. When the loading continues, three stress values increase slightly, and the crack in the slope penetrates to form an apparent fracture surface. At this time, the slope will be extensively damaged in a large scale (see Fig. 8e). The deformation of constant resistance bolt has been further increased, and it has been destroyed at last. It plays an integrated role of tensile, monitoring and early warning.

Using AE technology to study initiation and propagation of microcrack in rock is helpful for comprehensively and truly understanding the process of rock failure and deformation^33–35^. The size of the circle in AE diagram of this paper represents the relative energy or magnitude, which is proportional to the strength of the element: a white circle represents a shear failure, while a red circle represents a tensile failure^36^. According to the AE diagram, the tension failure of the slope first occurs under centrifugal loading, and cracks begin to appear at the foot of the slope. At the same time, the initial tension crack extends to the deep part of the slope under the condition of continuous loading, and shear failure occurs gradually in the AE mode on the basis of tension failure. Finally, instability failure occurs in the slope under the combined action of tension and shear.

### Point P displacement analysis

**Figure 9:**
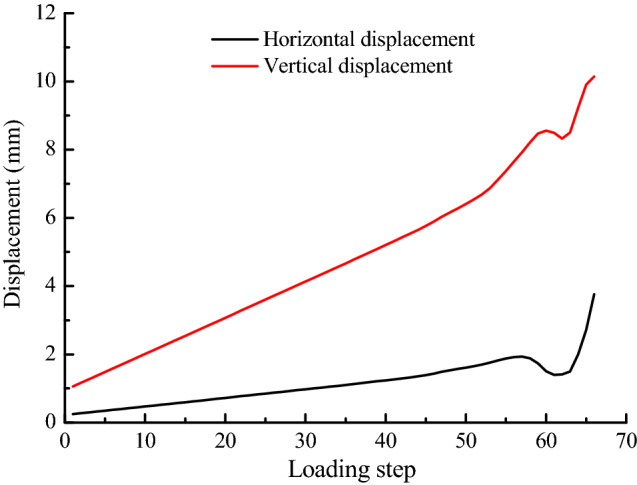
Point P displacement curve of landslide process.

It can be seen from Fig. 9 that the horizontal displacement and vertical displacement increase linearly with the increase of loading step in the initial stage of loading, and the displacement and stress appear the same trend. In addition, the rate of increase of vertical displacement is greater than the rate of increase of horizontal displacement. As the loading continues, the increase rate of horizontal and vertical displacements is obviously greater than that of initial loading stage. The slope has shown obvious strain localization during this period, and the obvious cracks appear in the slope below the constant resistance bolt (see Fig. 8b). When loading continues, the horizontal displacement has a minimum value at step 61, the vertical displacement has a minimum value at step 62, both of which are prior to the stress. Strain localization and stress concentration gradually expand upward at present, cracks are generated in the upper slope of the constant resistance bolt, and the deformation of this constant resistance bolt has increased. After two displacement curves are lowered, they continue to rise. In the process, cracks appearing at the foot of slope penetrate through the entire slope, which cause slope overall instability failure. Simultaneously, the constant resistance bolt has been broken. When the slope is completely broken, the point P is horizontally displaced to the right to a maximum of 2 mm, and the vertical downward displacement is up to a maximum of 9.24 mm.

### Numerical model AE distribution

**Figure 10:**
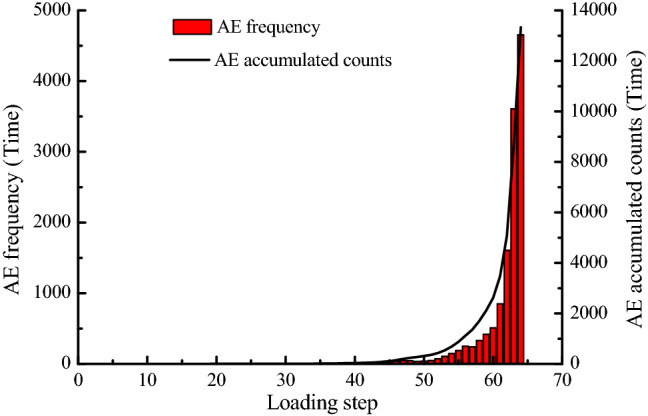
AE curve of numerical model.

Figure 10 is a graph showing the relationship between AE frequency and AE cumulative number of numerical model with the loading obtained by numerical experiments. The numerical experiment has shown that although there are some AE phenomena in the early stage of loading, cumulative amount of acoustic emissions are small. The number of acoustic emissions has increased significantly from the 60th step, increasing by 358, and the number of AE increased greatly after each loading step. The number of acoustic emissions of the five loading steps accounts for 84.19% of the total number from step 60 to 64, indicating that AE phenomenon mainly occurs in the late stage of the slope failure. Comparing the stress-loading step curve, it can be seen that when the stress starts to decrease from the maximum value, AE curve begins to show a large rise.

### Results discussion

The important basis is the whole process of slope internal stress and displacement changes in the monitoring and forecasting of landslide geological disasters. It mainly includes three stress components and two displacement components for numerical calculation of plane strain. In this paper, the whole variety process of three stresses and two displacements of point P on bolt in the slope have been simulated by RFPA-Centrifuge software. Combining the elastic modulus diagram and AE diagram of the landslide process, it is known that the maximum principal stress, minimum principal stress and shear stress of point P before the overall failure of slope are abruptly increased and suddenly decreased, and both the horizontal displacement and vertical displacement curve have a process of ascending and descending. At the same time, the AE curve has a large increase from a certain loading step compared to the previous period.

Point P Newton force $F = \sigma \times A$, $\sigma$ is stress, *A* is area of stress action surface. Force and stress are vectors, and bolt have the identical changing trend. M.C. He two-body catastrophic mechanics theory reveals a phenomenon that Newton force decreases on the two-body interaction surface (landslide surface) in the process of landslide. In this paper, numerical experiment is carried out to obtain the phenomenon that stress and displacement on the landslide surface decrease, above two phenomena are essentially identical.

The loading step in RFPA-Centrifuge numerical experiment corresponds to the time variable in indoor physical experiment and field monitoring, there is a time interval between the two loading steps. This numerical test shows that when the stress and displacement suddenly drop to a minimum value, there is no overall instability failure of the slope, but the crack penetrates to the slope top in the next loading step (loading time), and the slope is destroyed completely. That is, the stress and displacement sudden drop does not represent the disintegration of landslide. In other words, the landslide disintegration occurs at a certain time interval after the sudden drop of stress and displacement. This is consistent with the conclusion that the time of Newton force catastrophe confirmed by M.C. He is the mechanical imbalance time of landslide double blocks. From the mechanical imbalance to the landslide disintegration, the conclusion that the time interval of at least 4–8 h is needed is consistent.

The occurrence of landslide can be predicted by the changing process of stress and displacement curves. When both curves reach the minimum point from the maximum point, the instability of the slope is predicted, that is, “stress and displacement sudden drop, catastrophic occurrence”. In this case, seasonable response measures should be taken according to the site conditions, or personnel and property should be immediately transferred to a safe place, or the slope should be immediately reinforced to prevent landslides.

## Conclusion

1. The whole process of slope failure with constant resistance bolt have been numerically calculated by using RFPA-Centrifuge software. The results show that the maximum principal stress, the minimum principal stress and the shear stress at point P have experienced a sudden drop and a sudden rise. The quantity of AE rises sharply in the case of sudden stress dropping, and the number of AE reaches its maximum value when the slope is destroyed as a whole. In addition, these three kinds of stress drop values are greater than stress rise values, disaster occurs after stress drop.
2. The horizontal displacement and vertical displacement curve of point P on the constant resistance bolt have a process of ascending and descending, the rise and fall of displacement curve is earlier than that of stress curve, and the change range of displacement curve is less than the stress curve. Disasters occur after the sudden drop of displacement.
3. The constant resistance bolt has a large plastic strain after slope failure, and there is a rupture. According to the AE diagram, the slope failure mode is the combined tension and shear failure.
4. The early warning of slope imminent sliding has always been a difficult and hot issue in the study of geological disasters. The conclusion drawn in this paper through numerical simulation is consistent with the field practice, that is, inserting a constant resistance bolt into the slope can realize landslide early warning. This conclusion provides a new idea for landslide monitoring.
